# Real-Time and Offline Evaluation of Myoelectric Pattern Recognition for the Decoding of Hand Movements

**DOI:** 10.3390/s21165677

**Published:** 2021-08-23

**Authors:** Sara Abbaspour, Autumn Naber, Max Ortiz-Catalan, Hamid GholamHosseini, Maria Lindén

**Affiliations:** 1 Department of Neurology, Massachusetts General Hospital and Division of Sleep Medicine, Harvard Medical School, Boston, MA 02114, USA; 2 Center for Bionics and Pain Research, 431 80 Möndal, Sweden; anaber@pm.me (A.N.); maxo@chalmers.se (M.O.-C.); 3 Department of Electrical Engineering, Chalmers University of Technology, 412 96 Gothenburg, Sweden; 4 Operational Area 3, Sahlgrenska University Hospital, 431 80 Mölndal, Sweden; 5 Department of Orthopaedics, Institute of Clinical Sciences, Sahlgrenska Academy, University of Gothenburg, 431 80 Mölndal, Sweden; 6 Department of Electrical and Electronic Engineering, Auckland University of Technology, Auckland 1010, New Zealand; hamid.gholamhosseini@aut.ac.nz; 7 School of Innovation, Design and Engineering, Mälardalen University, 722 20 Västerås, Sweden; maria.linden@mdh.se

**Keywords:** classification, electromyography, hand movement, pattern recognition, real-time

## Abstract

Pattern recognition algorithms have been widely used to map surface electromyographic signals to target movements as a source for prosthetic control. However, most investigations have been conducted offline by performing the analysis on pre-recorded datasets. While real-time data analysis (i.e., classification when new data becomes available, with limits on latency under 200–300 milliseconds) plays an important role in the control of prosthetics, less knowledge has been gained with respect to real-time performance. Recent literature has underscored the differences between offline classification accuracy, the most common performance metric, and the usability of upper limb prostheses. Therefore, a comparative offline and real-time performance analysis between common algorithms had yet to be performed. In this study, we investigated the offline and real-time performance of nine different classification algorithms, decoding ten individual hand and wrist movements. Surface myoelectric signals were recorded from fifteen able-bodied subjects while performing the ten movements. The offline decoding demonstrated that linear discriminant analysis (LDA) and maximum likelihood estimation (MLE) significantly (*p* < 0.05) outperformed other classifiers, with an average classification accuracy of above 97%. On the other hand, the real-time investigation revealed that, in addition to the LDA and MLE, multilayer perceptron also outperformed the other algorithms and achieved a classification accuracy and completion rate of above 68% and 69%, respectively.

## 1. Introduction

Electromyography (EMG) can provide a control source for prosthetic limbs by mapping activity from residual muscles to control signals. However, the complexity of this signal introduces challenges to its application, including the counter-intuitive nature of many control algorithms [1]. EMG-based pattern recognition could enable more intuitive control of powered prosthetics by learning and interpreting features, extracted from multichannel EMG signals, to predict motor intention directly.

Many studies have investigated the offline performance of the pattern recognition algorithms with different combinations of preprocessing, feature extraction, dimensionality reduction, and classification by analyzing pre-recorded signals. Often, these works present high offline classification accuracies for decoding motor intention [2]. Englehart et al. [1] used linear discriminant analysis (LDA) and multilayer perceptron (MLP) to decode the time domain (TD) and time-frequency domain features of four movements, from two-channel EMG signals, achieving an accuracy of up to 93.7%. Oskoei et al. [3] evaluated several feature sets, including the Hudgins set [4], comprised of mean absolute value (MAV), zero crossings (ZC), slope sign changes (SSC), and waveform length (WL), using artificial neural network and LDA classifiers and achieved 98.12% accuracy, with 6 features from 4 channels. Phinyomark et al. [5] investigated the performance of several individual features and feature sets using a support vector machine (SVM) to decode hand gestures, using EMG signals, available in different datasets with classification accuracies up to 95%. Although these offline studies have shown that the accurate decoding of gestures from electrodes placed on the forearm can be achieved, the questions of the controllability and classification accuracy of these systems for use in real-time (<300 ms) continuous classification have been primarily left open [6]. The obtained offline results might be well suited to some applications, but do not necessarily translate to systems useful for effective treatment of phantom limb pain in amputees, effective control of hand prostheses, and opportunities for improved motor rehabilitation after stroke and spinal cord injury. Therefore, further investigation is required to corroborate the translation of these findings to real-time performance [7].

A limited investigation has been performed regarding the real-time performance of pattern recognition algorithms. Guo et al. [8] compared the performance of ANN and SVM using four feature extraction methods in a seven–subject study. Their results on six surface EMG channels showed that SVM performed better in real-time, with an average accuracy of 85.9%. Moreover, Wang et al. [9] conducted a study on four healthy subjects to decode eight finger movements from surface EMG. Using results from offline tests, they then performed real-time tests, using a set of six features with an SVM classifier, and achieved accuracies between 90–100% when subjects were presented with visual feedback. Ortiz-Catalan et al. [10] reported the offline and real-time classification accuracy of LDA, MLP, and regulatory feedback networks (RFN) using the Hudgins set to decode ten movements. Average classification accuracies of 92.1% and 67.4% were obtained for offline and real-time tests, respectively. Two sets of experiments (offline and real-time) were performed by Krasoulis et al. [11] on 22 (20 able-bodied, 2 amputees) and 12 (11 able-bodied, 1 amputee) subjects, respectively. Four time-domain features, together with the LDA classifier, were used for the offline and real-time decoding of hand motions using 12-channels EMG signals. The highest offline classification accuracies of 82.7% (for able-bodied subjects) and 77.8% (for amputee subjects) were obtained using surface EMG and inertial measurements for the decoding of 40 different motions. The completion rate of above 90% (for able-bodied subjects) and 80% (for amputee subjects) were obtained in real-time for a sub-set of six classes. Geng et al. [7] investigated the offline and real-time performance of different classifier configurations in reducing the impact of arm position variation. Hodgins set was used as the feature set and LDA, with three classifier configurations, was used as the classifier. The study, across seven classes of movements, in five different arm positions, provided an average offline accuracy rate of 83.60% to 93.30% and a real-time completion rate of 64.60% to 77.30% for the three classifier configurations. Jiralerspong et al. [12] employed spectral features and an ANN for the real-time classification of different hand movements using six-channel EMG signals. The proposed algorithm achieved a real-time classification accuracy of up to 83% for 17 voluntary movements and 92% for 9 motions, across 12 subjects. These studies illustrated that offline accuracy does not have a good correlation with real-time performance and, in fact, can be a misleading metric to evaluate the usability of decoding algorithms in real-time applications [13].

Most of these studies evaluated a small number of classification algorithms, mainly ANN, LDA, SVM, and KNN. To the best of our knowledge, Scheme et al. [2] presented the only work that investigated a wide range of classification algorithms offline. They used the Hudgins set to classify 11 classes of motion using 10 common classification techniques. The one-versus-one classifier significantly outperformed all others, with an offline classification accuracy of 90.9%. Since offline accuracy provides an incomplete picture of how well the algorithms perform during continuous real-time classification, we built on this work to include additional classifiers and real-time performance metrics. A literature review was performed to identify the classification algorithms most often cited in published work with promising offline results. We investigated both the offline and real-time classifications of ten hand and wrist movements using nine different classification algorithms (MLP, RFN, supervised self-organized map (SSOM), LDA, SVM, KNN, maximum likelihood estimation (MLE), decision tree (DT), and non-negative matrix factorization (NMF)), in combination with the Hudgins set [4]. The real-time test used in this study is a modified version of the motion test [14] that is often cited for real-time performance metrics and analysis; however, direct comparisons of different algorithms using a similar dataset are still underreported.

In addition to providing an experimental investigation on the real-time and offline classification performance of a large number of algorithms, in the current study, an inclusive set of performance metrics was employed to compare the classification performance. A classifier not only should be able to produce an accurate classification, but this must be made efficiently and within the time constraints needed for real-time control. Only a few studies have compared algorithms, with respect to their usability for real-time control [15].

## 2. Materials and Methods

### 2.1. EMG Data Acquisition

Surface EMG signals, representative of ten classes of motions, were collected from fifteen healthy subjects (ten males and five females), with an average age of 33.1 (S.D. ± 6.16) years, 76.8 (S.D. ± 15.3) kg weight, and 175.9 (S.D. ± 11.4) cm height. The ethics approval was obtained from the Regional Ethics Review Board in Uppsala, Sweden (protocol code 2018/084 and date of approval 7 March 2018) and written, informed consent was obtained from the participants of the study.

The motion classes consisted of hand open/close, wrist flexion/extension, forearm pronation/supination, side grip, fine grip, agree, and pointer (for figures of these hand movements please see Figure 1 in [16]). These movements were selected because they are feasible in the current generation of high-end commercial prostheses.

**Figure 1:**
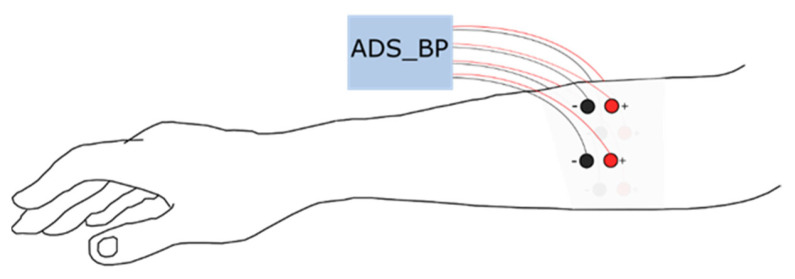
Surface electromyogram electrode position around the proximal third of the dominant forearm.

Eight disposable Ag/AgCl electrodes (diameter = 10 mm), in a bipolar configuration, were placed equally distributed around the proximal third of the dominant forearm; one distal and one proximal (see Figure 1). The first pair (channel 1) was placed along the extensor carpi ulnaris and the rest followed the lateral direction around the forearm. The proximal electrode was always connected to the positive terminal of the biopotential amplifier. Data were acquired using an open-source bioelectric signal acquisition system (ADS_BP) [17] with a high-pass filter with a cutoff frequency of 20 Hz, a low-pass filter with a cutoff frequency of 500 Hz, and a notch filter at 50 Hz. The recorded signals were amplified using a built-in gain amplifier (ADS1299) with a gain of 24 V/V. The signals were sampled at 1000 Hz with 24-bit resolution.

The data acquisition, offline analysis, and real-time testing were performed using BioPatRec software, a modular platform implemented in MATLAB that includes all the required functions for myoelectric control (see [10] for more details). To conduct this study, we made some modifications and implemented some additional algorithms (i.e., KNN, DT, MLE, and NMF) to the BioPatRec. This software ran on MATLAB R2014a.

During the recording sessions, subjects were instructed to perform the ten selected movements, guided with appropriate visual cues, such as images and progress bars (by BioPatRec software), to execute and hold each motion for a certain period of time and relax between each contraction. The subjects were asked to rest their elbow on an armrest during each movement [2]. Most of the subjects used BioPatRec for the first time (80%). None of the subjects had a history of neuromuscular disorders. In total, each subject performed twelve trials during the only recording session. The first and second trials were performed to train/prepare the subjects for offline and real-time tests. The recorded EMG during the third trial (offline trial) was used for the offline training/testing of the algorithms. In this trial, the subjects were asked to perform and hold each hand/finger movement for 3 s, followed by a relaxation of 3 s. This process was repeated three times, resulting in 18 s of data for each motion. The nine remaining trials (real-time trials) were performed using the motion test [10] to investigate the real-time performance of the nine classifiers (one trial for each classifier). For the real-time evaluation of the classifiers, each classifier was first trained using the recorded data in the offline trial. Then the real-time trial was used for real-time testing.

During the motion test, subjects were asked to execute the ten selected motions three times (in a randomized order). During the performance of a movement, the system was given up to 10 s to make 20 correct predictions. Subjects were given periods of rest between trials to avoid fatigue. The chosen hand and wrist movements for the real-time test were the same as for the offline tests. The implementation of the motion test in BioPatRec and further description can be found in [10]. The order in which the classifiers were evaluated, using the motion test, was randomized between subjects. The PC used for the offline and real-time tests was an HP running 64-bit Windows 10, with the processor at 2.90 GHz (Intel i7–7820, Intel, Santa Clara, CA, USA), and 16 GB of RAM.

### 2.2. Preprocessing

The recorded EMG signal during each movement (the 3 s contraction time) was trimmed equally at the beginning and end of the contraction time (15% each) to exclude inactive periods during the contraction. It was found empirically in [10] that the center 70% of contraction time was enough to partially conserve transient information. The transient periods were included for real-time control, which has been shown beneficial in some studies. Overlapping windowing of 200 milliseconds (ms), with 50 ms time increments, was used for signal segmentation, which means, for the real-time test, EMG processing and pattern recognition was applied every 50 ms, based on the previous 200 ms of data. It has been shown, through information theory, that EMG windows of 100 to 300 ms contain the highest information content [18]. The classification accuracy increases with longer window lengths, despite the corresponding increase in controller delay; therefore, both of these effects should be considered when choosing a window length. The optimal length, for this specific task, has been suggested to be between 150 and 250 ms [19,20], which is within acceptable controller delays for conventional multi-state amplitude controllers [20].

### 2.3. Feature Extraction

In feature extraction, the most commonly used set of features (four TD features, reported by Hudgins et al. [4]: MAV, ZC, SSC, and WL) was employed over each of the four channels to produce a feature set with 16 dimensions.

- Mean Absolute Value (MAV)

MAV is obtained by averaging the absolute value of the EMG signal in a window [21]. A large increase occurs in the value of this feature at onset and remains high during the contraction [22].

- Zero Crossings (ZC)

ZC counts the number of times that the sign of the amplitude of the signal changes [23].

- Slope Sign Changes (SSC)

SSC represents the frequency properties of the EMG signal, and it counts the number of times the slope of the EMG signal in a time window changes sign [24].

- Waveform Length

Waveform length of the signal gives information about the complexity of the signal in a window by summing the numerical derivative of the sample window [25].

### 2.4. Classification

**Figure 2:**
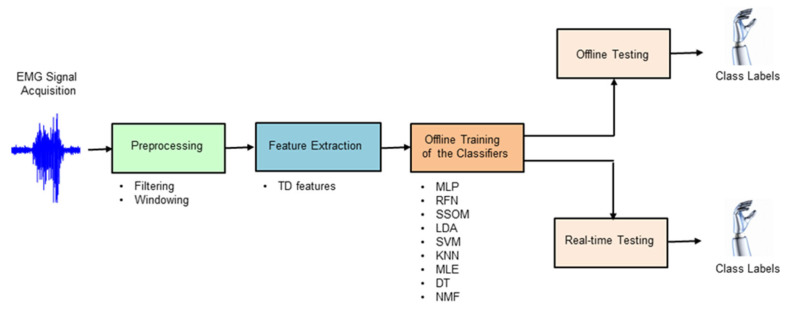
Block diagram illustrating the processing stages used in this study to decode hand movements, both offline and in real-time.

Classifier-specific feature normalization was used to ensure numerical stability for the tested classification algorithms, as it has been shown to significantly affect classifier performance [10]. Nine different classification techniques (MLP, RFN, SSOM, LDA, SVM, KNN, MLE, DT, and NMF) were chosen in this study by extensively reviewing the state of the art (see Section 2.4.1, Section 2.4.2, Section 2.4.3, Section 2.4.4, Section 2.4.5, Section 2.4.6, Section 2.4.7, Section 2.4.8 and Section 2.4.9 for more details about the classifiers). The criterion for inclusion was the most cited algorithms in published works with promising results. Figure 2 shows the processing stages of offline and real-time myoelectric pattern recognition for decoding ten hand movements.

To train, validate, and test the classifiers, the feature vectors were divided into 40% for training, 20% for validation, and 40% for testing. Training and validation data were used in training the selected algorithms and viewing preliminary offline results. The testing set was not presented to the classifiers during the training process but was used to compute offline accuracy after training was completed. Cross-validation was performed ten times by randomizing the feature vectors in the training, validation, and testing sets [26].

#### 2.4.1. Multilayer Perceptron (MLP)

A feedforward multilayer perceptron network with 16 input neurons (4 features × 4 channels), two hidden layers (of 16 neurons each), and ten output neurons (one neuron per movement) was used for classification in this work. The activation function, in all neurons, was logistic (sigmoidal). The learning method used was backpropagation, with the learning rate and momentum of 0.1. The training was stochastic, with a maximum of 200 iterations for convergence. The 0-midrange with 2-range (−1 to 1) normalization method, implemented in BioPatRec, was used for the MLP classifier, as it was empirically found in [10] that this type of normalization reduces the training time and improves the convergence of the MLP.

#### 2.4.2. Regulatory Feedback Networks (RFN)

The RFN is a relatively new instance in pattern recognition that performs based on negative feedback [10]. Negative feedback from the previous prediction is used to dynamically train the neurons’ weights as it makes new predictions [27]. The unitary range (0 to 1) normalization method was preferred for the RFN classifier [10].

#### 2.4.3. Supervised Self-Organized Map (SSOM)

SSOM is a type of artificial neural network that works based on competitive learning [28]. SSOM is considered one of the most robust learning strategies among neural network algorithms [29]. There were several parameters for SSOM in BioPatRec that needed to be set: training method, grid shape, and neighbor function; in this study, these parameters were set to stochastic, rectangular, and Gaussian, respectively, as they were chosen as the default parameters in BioPatRec, due to their broad range of applicability. The norm-log (x ≥ 0) normalization method was used here, since it is preferred for the SSOM classifier [10].

#### 2.4.4. Linear Discriminant Analysis (LDA)

LDA is a simple, efficient, and statistically-based classifier that has been used extensively in prosthetic control, due to its high performance in the classification of EMG signals, the robustness in long-term effect usage, and the low computational cost [24]. The discriminant analysis algorithm (type: linear), provided by MATLAB R2014a, with one-vs-one (OVO) topology was used to classify the 11 hand motions. In an OVO topology, an individual classifier is trained to discriminate between each combination of two movements. The output is computed by majority voting, and therefore, can only have one winning class for each prediction in this configuration [13]. It has been observed in [10] that there is no need to normalize the data when using LDA.

#### 2.4.5. Support Vector Machine (SVM)

SVM is a viral machine learning algorithm that uses kernels to map data into separable hyper-planes [2,30]. In this study, the kernel type was set to polynomial [31]. The 0-midrange with 2-range (−1 to 1) normalization method was empirically found to be a good choice for the SVM classifier.

#### 2.4.6. K-Nearest Neighbor (KNN)

In this study, Euclidean distance was chosen as the distance metric. In choosing K in the KNN classifier [32], it was observed that a larger value of K decreases the classification accuracy. We carried out our experiments with different values of K: 1 to 10, 40, and 100; K = 1 provided the highest classification accuracy. We integrated the k-nearest neighbor algorithm, provided by MATLAB R2014a in BioPatRec. The norm-log (x ≥ 0) normalization method was used, as it is preferred for the KNN classifier [10]. 

#### 2.4.7. Maximum Likelihood Estimation (MLE)

The MLE algorithm finds the statistical parameters that most likely describe input data, given the model function [28,29]. In EMG classification, extracted features from each class, assumed to be independent and normally distributed, are fed into a Gaussian MLE function to find the parameters (mean and covariance in this case) that best fit each movement to train the system. During testing, EMG features are fed into all MLE models and class, with the highest probability selected as the output. We implemented the MLE algorithm from [33] in BioPatRec. Different normalization methods were tested for the MLE classifier, but they did not show any impact on the performance of this algorithm, so no normalization was used. 

#### 2.4.8. Decision Tree (DT)

In EMG classification, DT uses a set of features extracted from the signals to form a decision [34,35]. To perform the DT classification in our experiments, we integrated the included algorithm from MATLAB R2014a into BioPatRec. Normalization methods did not show any impacts on the performance of the decision tree, likely because DT does not make any assumptions about the shape of the data.

#### 2.4.9. Non-Negative Matrix Factorization (NMF)

NMF is a matrix decomposition method that decomposes a non-negative matrix into two low-rank, non-negative matrices. This method has been successfully used for biological data mining, as well as clustering [36]. To implement this algorithm in BioPatRec, the non-negative factorization matrix toolbox was integrated [36]. There are several classifiers available in the NMF toolbox, and non-negative least squares (NNLS) was chosen in this study, due to its time performance. The NNLS classifier can be used to classify under-complete data (data that contains very few numbers of samples, compared to the feature set). The NNLS algorithm works by treating the training samples as basis vectors and minimizing the squared difference between the new sample and the non-negative linear combination of the basis matrix and coefficient vector. The class label is taken from the coefficient with the largest value [37]. This corresponds to the closest sample in the column space of the basis matrix. The 0-midrange with 2-range (−1 to 1) normalization method was empirically found to be a right choice for this classifier.

All the parameters in this study (parameters of the classifiers, the length of the training/validation/testing data, etc.) were selected, based on previous studies in BioPatRec [10,16].

### 2.5. Evaluation

The recorded signals of the third trial of each subject were used to evaluate the offline performance of the classifiers. The offline performance was evaluated using accuracy rate, training time (in seconds), and testing time (in ms). To evaluate the real-time performance of the classifiers, the real-time accuracy, selection time (in seconds), completion time (in seconds), and completion rate were used as the performance indicators. Real-time accuracy was calculated as the recall (percent of correct predictions over total predictions) between the first (non-rest) prediction and the 20th correct prediction. Selection time is the time between the first prediction (different than ‘rest’) and the first correct prediction. Completion time is the time between the first prediction (different than ‘rest’) and the 20th correct prediction. The completion rate is the percentage of movements that reached 20 percent correct predictions before timeout [26].

To show the significant differences between classifiers’ performance, in both offline and real-time, the performance metrics were analyzed as a completely randomized design with one-way analysis of variances (ANOVA), using the general linear model procedure of the SAS software (SAS Institute Inc. 2004). Duncan’s multiple range test was used to test the significance of the differences between means. All significances were declared at *p* < 0.05. The data was found to be sufficiently normal for use in ANOVA tests by visual investigation of the QQ-plot.

## 3. Results

### 3.1. Accuracy

Table 1 summarizes the offline and real-time performance of each classifier. LDA and MLE obtained the best offline accuracy, with averages of 97.9% and 97.0%, respectively. The differences were statistically significant (*p* < 0.05) against the other classifiers. MLP and DT obtained the next highest offline accuracies at 91.7% and 92.2%, respectively, which were statistically significant against the other classifiers (*p* < 0.05). SSOM and KNN obtained the lowest accuracy rates, with statistically significant results (*p* < 0.05).

The MLE classifier required the least amount of time for training and validation, at an average of 0.171 s. The training and validation process for RFN, SSOM, LDA, SVM, KNN, and DT classifiers took an average of 0.442, 5.85, 0.342, 0.679, 0.507, and 0.408 s, respectively. The differences were statistically significant (*p* < 0.05) against the MLP and NMF classifiers, with average training times of 14.9 and 46.3 s, respectively. NMF was the most time-consuming classifier for the training (*p* < 0.05). The fastest algorithm for offline testing was MLP, with an average of 0.115 ms. The testing times for the RFN, SSOM, LDA, SVM, KNN, MLE, and DT classifiers averaged 0.935, 0.162, 2.20, 2.43, 9.69, 0.150, and 0.642 ms, respectively. The differences were statistically significant (*p* < 0.05) against NMF, the slowest algorithm in offline testing, with an average of 286.6 ms.

Based on the real-time accuracy obtained by the motion test, the MLP classifier obtained the highest accuracy rate, with an average of 69.8%. The difference was not statically significant against LDA and MLE, with average accuracies of 69.3% and 68.4%, respectively. However, the accuracy rate obtained by MLP was significantly (*p* < 0.05) better than that of the RFN, SSOM, SVM, KNN, DT, and NMF classifiers, with average accuracies of 60.9%, 35.5%, 43.0%, 33.3%, 59.8%, and 47.6%, respectively. No statistical significance was found between MLE, RFN, and DT; however, LDA performed better than DT (*p* < 0.05). The KNN classifier had the lowest accuracy rate (with an average of 33.3%), which was statistically significant (*p* < 0.05) against all the other classifiers (excluding SSOM).

**Figure 3:**
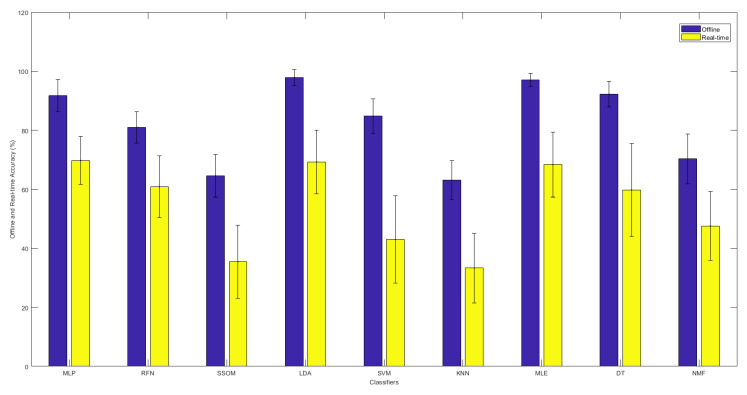
The offline and real-time average accuracies between classifiers, with their standard deviations in the top-center of each bar over 15 subjects.

Figure 3 illustrates the comparison between the offline and real-time accuracy rates of the classifiers. It can be seen in this figure that the offline accuracy of each classifier is always higher than its real-time accuracy. As was presented before, LDA and MLE had the highest offline accuracy rates. However, the highest real-time accuracy was found with the MLP classifier, though the real-time results of LDA, MLE, and MLP showed no statistical difference.

**Figure 4:**
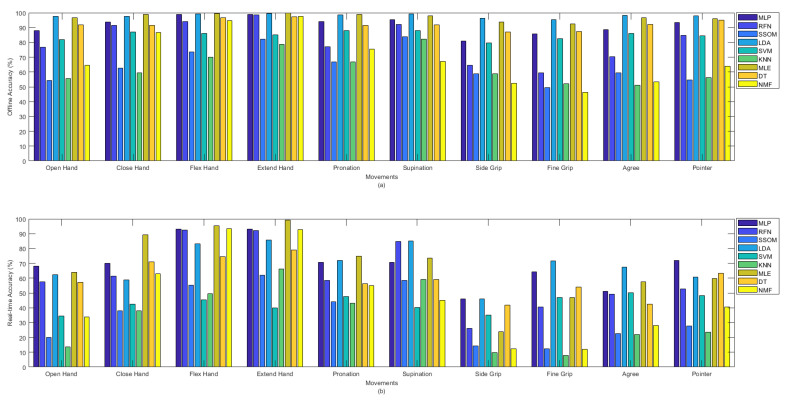
(**a**) Offline and (**b**) real-time average accuracies over 15 subjects between classifiers per movements.

Figure 4a shows the offline and Figure 4b shows the real-time accuracies, between classifiers, per movement. The presented offline accuracy in Figure 4a, shows that ‘extend hand’ was the easiest movement to predict, as most of the classifiers (e.g., MLP, RFN, LDA, MLE, DT, and NMF) were able to detect this movement better than the other movements. The second easiest movements to detect were ‘supination’ and ‘flex hand’. It is likely that there are less similarities between these movements and the other movements performed in this study. The more similarity between movements, the more likely that there is confusion for classifiers in discriminating different movements. By comparing the accuracy rates of different classifiers per movement in Figure 4a, it can be seen that the MLP, LDA, MLE, and DT classifiers obtained high accuracies in detecting almost all the movements in the offline evaluation. Based on the obtained real-time accuracy by different classifiers for each movement in Figure 4b, ‘flex hand’ and ‘extend hand’ were the easiest and ‘side grip’ was the hardest movement to detect. Most of the classifiers had low performance in detecting ‘side grip’. The real-time accuracies in Figure 4b showed that among all the classifiers, the MLP, LDA, MLE, and DT obtained higher accuracies in detecting all the movements (the same as what had been seen in Figure 4a for the offline accuracies).

**Figure 5:**
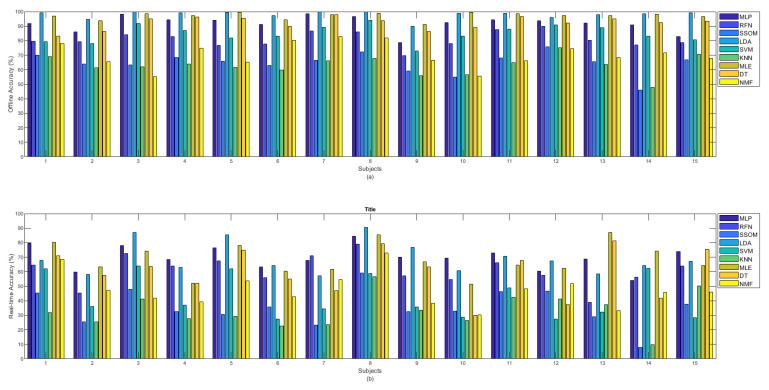
(**a**) Offline and (**b**) real-time accuracies between classifiers per subjects.

Figure 5a shows the offline and Figure 5b shows the real-time (b) accuracies between classifiers per subject. It can be observed from Figure 5a that among all classifiers, the MLP, LDA, MLE, and DT obtained the highest offline accuracy rates for all the subjects. Considering the obtained accuracies by all the classifiers, subject number 8, who was the most experienced in BioPatRec (both the movements and the EMG recording stages), produced the best result; however, the difference was small, compared to some other subjects. Subject numbers 1 and 15 had also used BioPatRec before, but it is difficult to see a big difference between their produced offline accuracies and that of the other subjects. Subject number 12 is a rock climber who has very strong forearm muscles. This subject’s offline accuracy for the RFN, SSOM, and KNN classifiers, interestingly, increased and obtained the highest accuracy rates among the other subjects.

The real-time accuracy, obtained by each subject in Figure 5b, illustrates that MLP, LDA, and MLE achieved the highest accuracy rates for ten subjects (subject numbers 2, 3, 5, 6, 8 to 12, and 15) out of 15. The real-time accuracy rates (obtained for all the classifiers) by the subject number 8, who was the most experienced in BioPatRec, were the highest, compared to the other subjects. Although the offline accuracy achieved by subject number 12 (the rock climber) showed a slight improvement in the SSOM and KNN, the real-time accuracy did not show any specific difference, compared to that of the other subjects.

During the data acquisition of subject number 14, it was observed that, despite filtering the signal with a notch filter at 50 Hz, the power-line noise still affected the signal. As it can be seen in both bar plots in Figure 5, this subject obtained the lowest offline and real-time accuracy rates for the SSOM and KNN classifiers. Based on this, it is likely that these classifiers are not very robust against baseline noise, though it is difficult to make a definitive statement based on only one subject.

### 3.2. Selection Time

**Figure 6:**
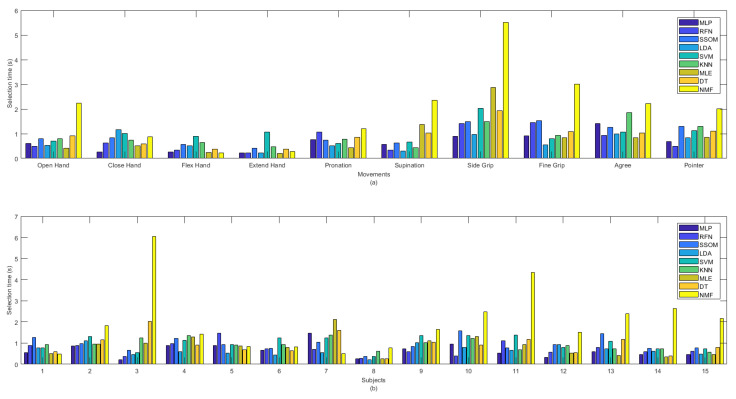
Selection time in second between classifiers per (**a**) movements and (**b**) subjects.

MLP obtained the lowest selection time (0.657 s), which was not statistically significant, against the other classifiers, but NMF had the highest selection time, with an average of 1.99 s (see Table 1). The selection time (in seconds) of all the classifiers, per movement (a) and subjects (b), are illustrated in Figure 6. The ‘extend hand’ movement showed the lowest selection time among all the other movements and SVM was the slowest classifier for this movement among the other classifiers. The ‘side grip’ movement had the highest selection time among all the other movements, with MLP as the fastest and NMF as the slowest classifier for this movement among all the other classifiers. By looking at the selection time for each subject in Figure 6b, it can be seen that subjects’ practicing the movements (subject number 8) improved not only the classification accuracy but also decreased the selection time.

Considerable variability was found among different movements and subjects for the selection time obtained by the NMF classifier. NMF captured the lowest selection time, among all the classifiers, for ‘flex hand’ and the highest selection time for ‘side grip’. This classifier also obtained the lowest selection time, among the other classifiers, for subject numbers 1 and 7, as well as the highest selection time, with high variability, for subjects 3 and 13.

### 3.3. Completion Time

**Figure 7:**
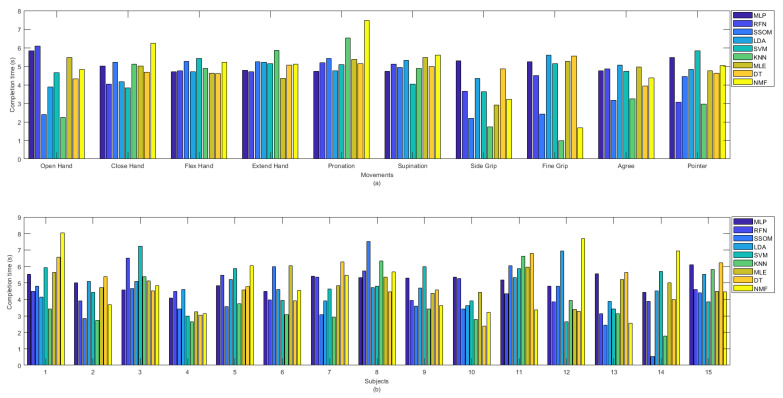
Completion time in second between classifiers per (**a**) movements and (**b**) subjects.

The KNN classifier achieved the lowest completion time, with an average of 3.85 s. The differences were statistically significant (*p* < 0.05), compared to all the classifiers (excluding SSOM and RFN), with average completion times of 4.07 and 4.60 s, respectively (see Table 1). MLP had the highest completion time, with an average of 5.06 s, and this was statistically significant against the KNN and SSOM classifiers (*p* < 0.05). No statistical significance was found between the completion times of the other classifiers. The completion time (in seconds) for all the classifiers per movement (a) and subjects (b) are presented in Figure 7. It can be observed that ‘side grip’ and ‘fine grip’, among the other movements, obtained the lowest completion time by most of the classifiers and KNN showed the lowest completion time. ‘Close hand’, ‘flex hand’, ‘extend hand’, ‘pronation’, and ‘supination’ obtained the high completion times, with low variability, among the classifiers. Because of the considerable variability among the classifiers, in Figure 7b, it is difficult to say which subject obtained the lowest completion time but, among all the classifiers, SSOM and KNN achieved the lowest completion times for subject number 14. Subject numbers 3, 8, and 11 obtained slightly higher completion time, compared to the other subjects.

### 3.4. Completion Rate

**Figure 8:**
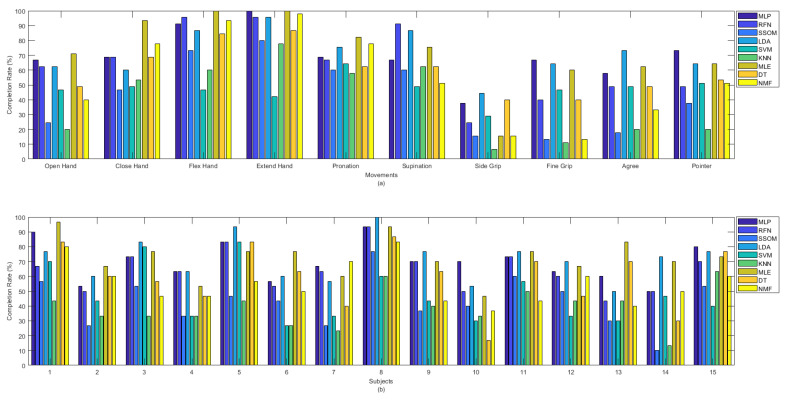
Completion rate between classifiers per (**a**) movements and (**b**) subjects.

Among all the classifiers, the highest completion rates were obtained by LDA and MLE, with average rates of 71.3% and 72.4%, respectively, which were statistically significant against SSOM, SVM, KNN, DT, and NMF (*p* < 0.05). SSOM and KNN obtained the lowest completion rates, and the differences were statistically significant (*p* < 0.05) against all the other classifiers (excluding SVM) (see Table 1). Figure 8 presents the completion rate of all the classifiers per movement (a) and subjects (b). The ‘flex hand’ and ‘extend hand’ movements had the highest completion rates among the other movements. The highest completion rate for these two movements was obtained by MLP, RFN, LDA, MLE, and NMF. SVM obtained the lowest rate for these movements. The ‘side grip’ movement had the lowest completion rate among the other movements, with the highest rate obtained by LDA and the lowest rate by KNN. Subject number 14 received the lowest completion rate for the SSOM and KNN classifiers (Figure 8b). Subject number 8, who was the most experienced in BioPatRec, obtained the highest completion rate for most of the classifiers.

## 4. Discussion

In this study, we compared the offline and real-time performance of nine different classification algorithms to detect ten individual movements, using four channels of surface EMG signals, recorded from 15 healthy volunteers. In addition to the accuracy rate, the offline performance was evaluated using training and testing time. To evaluate the real-time performance, several performance indicators were included from the motion test (accuracy and completion rate, as well as selection and completion time).

### 4.1. Offline and Real-Time Performance

Among all classifiers, LDA and MLE obtained the highest offline accuracy rate (above 97%) and completion rate (above 71%), with the lowest training time for MLE (0.171 s) (see Table 1). MLP obtained around 9% lower offline accuracy than LDA and MLE, while achieving the highest real-time accuracy rate of 69.8%, but suffered from a significantly (*p* < 0.05) higher training time (14.9 s). Although MLP showed the fastest testing and selection times, its completion time was the slowest. The offline accuracy obtained by RFN was significantly lower than that of DT (81.0% vs. 92.2%). However, the obtained real-time accuracy of RFN was numerically higher than that of DT (60.9% vs. 59.8%), though the difference was not statistically significant. The differences in their training and testing times were not statistically significant either, but DT was faster in both testing and training. Although the differences in the obtained offline accuracy rates between SVM and RFM were not significant (84.8% vs. 81.0%), RFN obtained a significantly higher real-time accuracy rate than SVM (43.0% vs. 60.9%). The differences in their training and testing times were not statistically significant, but RFN was faster in both testing and training. NMF obtained around 8% lower offline accuracy than SVM but achieved slightly higher real-time accuracy (43.0% vs. 47.6%). However, NMF was the slowest classifier in both training and testing. Its selection time was also the highest among the other classifiers. The reason for NMF being slow is that it does not train the classifier in the training phase, in the traditional sense, but computes a least-squares solution every time a prediction is made. The obtained offline and real-time accuracy rates by SSOM and KNN were significantly (*p* < 0.05) lower than that of the other classifiers. However, KNN had the lowest completion time.

### 4.2. Trends in Offline and Real-Time Performance Metrics

Less variability was found in detecting different movements in offline classification accuracy rates than in real-time (see Figure 4). Similarly, Figure 5 illustrated that classifiers were more consistent in the offline recognition of movements per subjects. However, this consistency was not retained in real time. Most of the subjects obtained very similar offline classification accuracies, but their real-time accuracies were considerably different. The results in Figure 4, Figure 5, Figure 6 and Figure 7 showed that the movements that obtained higher real-time accuracy rates, such as ‘flex hand’ and ‘extend hand’, obtained lower selection times and higher completion rates but high completion times. Inversely, the movements that had lower accuracy rates, such as ‘side grip’ and ‘fine grip’, obtained higher selection times and lower completion rates but low completion times. The subjects that obtained higher real-time accuracy rates obtained higher completion rates but high completion time. Such a pattern was not found for the selection times. However, subject number 8, who obtained a higher real-time accuracy rate, had the lowest selection time among the other subjects.

### 4.3. Comparison with Existing Literature

Five papers, found in this study, made comparisons between offline and real-time performance. Guo et al. [8] and Ortiz et al. [10] both reported a significant decrease between offline and real-time classification accuracy, which corroborates the trend seen in this work. However, Wang et al. [9] saw an increase in accuracy with real-time results. This counter-intuitive finding may be a result of a ceiling effect (all values were close to 100%) and the comparatively large number of channels used in their experiment. 

Krasoulis et al. [11] and Geng et al. [7] used different performance metrics (classification accuracy (for offline) and completion rate (for real-time)) to compare the performance of only one classifier (LDA). Krasoulis et al. [11] reported a lower offline classification accuracy and a higher completion rate, compared to the present study. One reason might be a higher number of motions in offline (40 classes) and a lower number of motions in real-time (6 classes) that were classified. The reported offline classification accuracies for the LDA classifier were lower, compared to the current study. This might be a result of testing the algorithms on amputated subjects, which some studies have found lowers accuracy in this population [38].

Only one paper was found that reported the comparisons of selection time, completion time, and completion rate between different classifiers. Ortiz et al. [10] documented the above metrics for LDA, MLP, and RFN classifiers. Little difference was found between the classifiers, which corroborates what was found in the current study, since significant trends were found only in other classifiers.

Different computers will perform classifier training and testing calculations at different speeds, so it is useful to have multiple direct comparisons within a single study. Guo et al. [8], Ortiz et al. [10], and Shin et al. [32] all reported the testing and training times for the classifiers and features used in their works. Similar to the present study, MLP tended to train much slower than SVM but classified predictions much faster.

### 4.4. Effect of Classifier Properties on Performance Metrics

LDA and Gaussian MLE assume linear separability in the data. Their relative success, in both offline and online classification accuracy, implies that the signal features generated from isometric EMG contractions demonstrates sufficient linear separability for the application of these algorithms. MLP and RFN do not make assumptions about the shape or distribution of the input data and do not necessarily rely on the linear separability of the classes, but they can be prone to overfitting and bias (if given biased or insufficient data for learning). However, both MLP and RFN benefit from linearly separable classes, since the relationships between the data and the classes are easier to learn. Their relatively high offline and real-time classification accuracies imply that signal processing and feature extraction were successful in supplying enough unbiased data, given the distribution, to produce an effective classifier.

The relatively slow testing (classification) speed seen in SVM, KNN, and (most notably) NMF classifiers can explain why the selection time and completion rate were relatively low. Since each classification took much more time than the other algorithms, the first successful classification (selection time) was late, and fewer classifications were performed, giving less opportunity to achieve enough classification for success.

KNN, NMF, and SSOM all performed relatively poorly, in terms of offline accuracy. While offline accuracy may be misleading, the comparative performance of different classification algorithms was found to hold fairly well, resulting in them achieving the lowest real-time accuracies.

### 4.5. Sources for Bias and Variability

Part of the effects of these trends can be explained by the way in which the real-time metrics were calculated. Any movements that were not completed (resulting in a lower completion rate) were discarded from the accuracy and completion time metrics. This biases the rest of the metrics towards movements with high accuracy. As a result, none of the real-time metrics are fully representative of the classifier performance in isolation. The results do indicate that there is considerably more variability in the results of classification systems running in real-time than in those running offline with pre-recorded data. The presented visual feedback in the real-time tests may have also influenced the muscle patients’ contraction patterns in real-time, as users may have modified their posture or the strength of muscle contraction to drive the desired classification output. This would have an unpredictable effect on the included metrics, possibly increasing the variance of the results.

In future work, latency with algorithms implemented in an embedded system will be tested. User preferences in control algorithms will be investigated. The performance of deep learning algorithms will also be evaluated in offline and real-time. As performance metrics and testing methodologies for biofeedback applications mature, more direct comparisons (and stronger links) between offline and real-time evaluation metrics will be investigated.

## 5. Conclusions

The experimental result of the current study showed a significant difference (around 14%) between the highest classification accuracy rates obtained in offline and real-time (97.9% vs. 69.8%). We believe that it is possible to decrease this difference by having subjects practice the movements more. Based on the obtained classification accuracy, LDA and MLE significantly (*p* < 0.05) outperformed the other classifiers in offline tests. However, in real-time, MLP had the highest accuracy rate, but the differences were not statistically significant against LDA and MLE. The accuracy rate per movement illustrated that most of the individual movements could be classified well offline, using MLP, RFN, LDA, SVM, MLE, and DT, but several movements from the same muscle groups were not easy to detect in real time. MLP showed a low testing and selection time but produced the highest completion time. Its completion rate was high, as well, but not as high as LDA and MLE. NMF was the most time-consuming algorithm, based on the training, testing, and selection time. Consequently, MLP, LDA, and MLE, with high accuracy and completion rates and low processing time, are potentially more optimal for prosthetic control.

## Figures and Tables

**Table 1:** Average and standard deviation of offline accuracy rate, training time (in seconds (s)), and testing time (in millisecond (ms)); real-time accuracy rate, selection time (s), completion time (s), and completion rate for all classifiers over 15 subjects. The best results in each column are bolded.

	Offline			Real-Time			
	Accuracy (%)	TrTime (s)	TTime (ms)	Accuracy (%)	SelTime (s)	CompTime (s)	CompRate (%)
MLP	91.7 ^b^ ± 5.47	14.9 ^b^ ± 0.474	**0.115 ^b^ ± 0.066**	**69.8 ^a^ ± 8.16**	**0.657 ^b^ ± 0.327**	5.06 ^a^ ± 0.528	69.8 ^ab^ ± 12.8
RFN	81.0 ^c^ ± 5.25	0.442 ^c^ ± 0.044	0.935 ^b^ ± 0.047	60.9 ^bc^ ± 10.4	0.732 ^b^ ± 0.310	4.60 ^abc^ ± 0.895	64.2 ^abc^ ± 13.7
SSOM	64.6 ^e^ ± 7.20	5.85 ^c^ ± 0.235	0.162 ^b^ ± 0.039	35.5 ^ef^ ± 12.4	0.955 ^b^ ± 0.316	4.07 ^bc^ ± 1.69	42.9 ^e^ ± 16.5
LDA	**97.9 ^a^ ± 2.69**	0.342 ^c^ ± 0.058	2.20 ^b^ ± 0.090	**69.3 ^ab^ ± 10.8**	**0.659 ^b^ ± 0.238**	4.79 ^ab^ ± 0.815	71.3 ^a^ ± 14.4
SVM	84.8 ^c^ ± 5.84	0.679 ^c^ ± 0.154	2.43 ^b^ ± 0.139	43.0 ^de^ ± 14.8	1.00 ^b^ ± 0.327	4.75 ^ab^ ± 1.31	47.3 ^de^ ± 18.6
KNN	63.1 ^e^ ± 6.63	0.507 ^c^ ± 0.181	9.69 ^b^ ± 0.628	33.3 ^f^ ± 11.9	0.944 ^b^ ± 0.262	**3.85 ^c^ ± 1.48**	38.9 ^e^ ± 13.1
MLE	**97.0 ^a^ ± 2.25**	**0.171 ^c^ ± 0.036**	0.150 ^b^ ± 0.033	**68.4 ^abc^ ± 11.0**	0.858 ^b^ ± 0.486	4.82 ^ab^ ± 0.806	**72.4 ^a^ ± 13.3**
DT	92.2 ^b^ ± 4.33	0.408 ^c^ ± 0.151	0.642 ^b^ ± 0.037	59.8 ^c^ ± 15.8	0.926 ^b^ ± 0.462	4.78 ^ab^ ± 1.34	59.6 ^bc^ ± 20.4
NMF	70.3 ^d^ ± 8.49	46.3 ^a^ ± 29.5	286.6 ^a^ ± 161.5	47.6 ^d^ ± 11.7	1.99 ^a^ ± 1.52	4.88 ^ab^ ± 1.72	55.1 ^cd^ ± 14.0

^a, b,…^ means within a column not sharing a common superscript are significantly different (*p* < 0.05).

## Data Availability

The obtained ethical approval restricts the availability of the data. But the BioPatRec software is available for data analysis at https://github.com/biopatrec/biopatrec accessed on 12 August 2021.
